# Epidemiology, Characteristics, and Treatment Outcomes of *Mycoplasma pneumoniae* Pneumonia in Hospitalized Adults: A 5-Year Retrospective Cohort Study

**DOI:** 10.1093/ofid/ofaf380

**Published:** 2025-06-26

**Authors:** Karl Hagman, Anna C Nilsson, Magnus Hedenstierna, Johan Ursing

**Affiliations:** Department of Infectious Diseases, Sahlgrenska University Hospital, Gothenburg, Sweden; Department of Infectious Diseases, Institute of Biomedicine, Sahlgrenska Academy at University of Gothenburg, Gothenburg, Sweden; Department of Infectious Diseases, Skåne University Hospital, Malmö, Sweden; Department of Translational Medicine, Infectious Diseases Research Unit, Lund University, Malmö, Sweden; Department of Infectious Diseases, Danderyd Hospital, Stockholm, Sweden; Department of Infectious Diseases, Danderyd Hospital, Stockholm, Sweden; Department of Clinical Sciences, Danderyd Hospital, Karolinska Institutet, Stockholm, Sweden

**Keywords:** antibiotics, extrapulmonary, incidence, mortality, symptoms

## Abstract

**Background:**

This study aimed to describe the incidence rate, patient characteristics, treatments, and outcomes of adults hospitalized with *Mycoplasma pneumoniae* pneumonia.

**Methods:**

This retrospective cohort study included adults diagnosed with *M pneumoniae* pneumonia and admitted to emergency care hospitals in Stockholm County, Sweden, from 2013 to 2017. Patients were identified through positive *M pneumoniae* polymerase chain reaction and *ICD-10* code J15.7 (*M pneumoniae* pneumonia). Medical records were reviewed manually, and population data were extracted from statistical databases. Incidence rates were calculated, and treatment outcomes were analyzed using regression models.

**Results:**

A total of 747 adults with a median age of 42 (interquartile range [IQR], 33–55) years, of whom 55% (385/747) were male, were hospitalized with *M pneumoniae* pneumonia. The incidence rate was 8.5 cases per 100 000 person-years, peaking at 14.1 in 2016. Cough (95%) and fever (92%) were the most common symptoms, and 71% were hypoxemic at admission. Patients with severe disease had longer symptom duration at admission. In-hospital mortality was 0.4%, and 6% required intensive care unit admission. Median length of stay (4 [IQR, 2–6] days) was longer in patients treated with macrolides (+1.0 [IQR, 0.9–1.2] days; *P* < .001) and fluoroquinolones (+0.8 [IQR, 0.1–1.4] days; *P* = .03) compared to those treated with tetracyclines. The median fever duration was significantly longer (+0.3 [IQR, 0.1–0.6] days; *P* = .02) in patients treated with fluoroquinolones compared to those treated with tetracyclines.

**Conclusions:**

The study highlights the importance of timely and accurate treatment of *M pneumoniae* pneumonia. Tetracycline treatment was associated with better outcomes, suggesting they may be an effective first-line treatment option.

## Teaser

The study analyzed 747 adults hospitalized with *Mycoplasma pneumoniae* pneumonia, finding an incidence rate of 8.5 per 100 000 person-years. Patients with severe disease had longer symptom duration at admission. Tetracycline treatment was associated with better outcomes compared to macrolides and fluoroquinolones.

Understanding the epidemiological characteristics of *Mycoplasma pneumoniae* pneumonia is vital for informed diagnostic decisions and optimized treatment strategies, particularly given its recent epidemic reemergence after several years of absence [1]. *Mycoplasma pneumoniae* infections occurs both endemically and with epidemic peaks approximately every 4 years [2]. A meta-analysis of etiological pneumonia studies in both outpatients and inpatients reported that *M pneumoniae* accounts for 18% (95% confidence interval [CI], 9%–26%) of cases, with significant heterogeneity due to the infection's epidemiological nature [3]. Additionally, in studies from the United States, *M pneumoniae* was identified as the most commonly detected bacterial pathogen in children hospitalized with community-acquired pneumonia (CAP) and the second most common in adults [4, 5]. *Mycoplasma pneumoniae* was found in 2% (43/2272) of adults hospitalized with CAP, corresponding to an incidence rate of 5 cases (95% CI, 4–7) per 100 000 person-years (PY) [4]. Adults with *M pneumoniae* pneumonia were younger, had fewer comorbidities, and experienced better outcomes compared to CAP of other etiologies [6].

*Mycoplasma pneumoniae* infections were traditionally diagnosed using serological methods. However, faster and more accurate nucleic acid amplification test (NAAT)–based diagnostics have been introduced in recent decades and are now the most prevalent method [7, 8]. It remains unclear whether the increasing use of NAAT methods for diagnostics will alter our understanding of the epidemiology and patient characteristics of *M pneumoniae* infections.

Macrolide antibiotics are considered first-line treatment for *M pneumoniae* infections in many countries due to their low minimum inhibitory concentrations and good tolerability [9]. However, the efficacy of macrolide treatment has been compromised by the increasing prevalence of macrolide-resistant *M pneumoniae* [10]. Other commonly used classes of antibiotics include tetracyclines and fluoroquinolones. Although there are few head-to-head comparisons, a recent meta-analysis found no significant differences in effectiveness among these antibiotic classes [11]. Treatments such as β-lactam antibiotics are ineffective for *M pneumoniae* due to its lack of cell wall [12]. Adjunctive corticosteroid treatment of adults hospitalized with *M pneumoniae* pneumonia was recently reported to correlate with shorter fever duration, but not with shorter time to regression of hypoxemia or length of stay [13].

The aim of this study was to describe the incidence rate, patient characteristics, treatments, and outcomes of adults hospitalized with *M pneumoniae* pneumonia over a 5-year period after the introduction of NAAT-based diagnostic testing.

## METHODS

### Study Design, Setting, and Population

This retrospective cohort study included adults (≥18 years of age) with pneumonia caused by *M pneumoniae* and hospitalized at any of the 7 emergency care hospitals in Stockholm County, Sweden, during 2013 to 2017. The region represents a total source population of about 1.75 million adult inhabitants.

Study subjects were identified by positive *M pneumoniae* microbiological sampling (polymerase chain reaction [PCR] from airway samples or paired sera) taken at the study hospitals and by searching medical records for the *International Classification of Diseases, 10th Revision* (*ICD-10*) code J15.7 (*M pneumoniae* pneumonia). Airway samples were analyzed at 2 accredited regional clinical microbiology laboratories using multiplex in-house assays based on previously described primers and probes [14]. From 2014, 1 of the laboratories changed to the multiplex assay Anyplex Ⅱ RB5 Detection (Seegene, Seoul, South Korea). Medical records were manually screened by a study physician to determine if patients were eligible for inclusion, and relevant data were extracted. Patients were not included if there was significant missing data, they were not hospitalized, there was no microbiological evidence of *M pneumoniae* infection (lack of positive PCR or paired sera), there was another more probable etiology for their hospitalization, and if there was no evidence of pneumonia (lack of radiological or clinical signs).

Estimated partial pressure of oxygen in arterial blood (PaO_2_)/fraction of inspired oxygen (FiO_2_) at admission was calculated from peripheral oxygen saturation (SpO_2_)/FiO_2_ as described elsewhere [15]. Included patients were categorized as having mild (estimated PaO_2_/FiO_2_ >300) or severe (estimated PaO_2_/FiO_2_ ≤300) disease at admission. Effective antibiotic treatment was defined as any antibiotics from the fluoroquinolone, macrolide, or tetracycline groups.

### Statistical Analysis

Continuous variables were presented as medians (interquartile range [IQR]) and categorical variables as numbers (percentage). Differences between groups were analyzed using the Wilcoxon rank-sum test or Kruskal-Wallis test for continuous variables and Fisher exact test or χ^2^ test for categorical variables. The Spearman rank correlation coefficient was calculated for symptom duration and estimated PaO_2_/FiO_2_.

Population denominator demographic data including number of inhabitants in the region stratified by age and sex for all respective years of the study period were extracted from Statistics Sweden's Statistical Database for calculation of incidence rates. Data on the total number of bacterial pneumonia diagnoses (*ICD-10* codes J13–J16 and J18) in hospitalized patients during the study period were extracted from the Swedish National Board of Health and Welfare's National Patient Register.

Median regression models were fitted to investigate the correlation between class of antibiotic treatment during hospitalization, length of stay, and fever duration. Potential confounders (age, sex, estimated PaO_2_/FiO_2_, symptom duration, corticosteroid treatment, and time from admission to effective antibiotics) were included in multivariable analyses. The model allowed for clustering of patients in regards to the hospital they were admitted to. Patients who died or did not receive any effective treatment during hospitalization were excluded from this analysis. Sensitivity analyses were performed including only patients who did not switch to another antibiotic class during hospitalization. Kaplan-Meier curves stratified by disease severity were constructed to visually describe time to discharge for different classes of antibiotics. The log-rank test was used to evaluate the equality of the survivor function across groups.

A *P* value <.05 was considered significant. Statistical analyses were performed using STATA-SE version 18.0 software (StataCorp LLC, College Station, Texas, USA).

### Patient Consent Statement

The Regional Ethical Review Board in Stockholm, Sweden, approved of the study (registration number 2018/797-31). Informed consent was waived due to the retrospective nature of the study.

## RESULTS

### Baseline Characteristics

**Figure 1.**
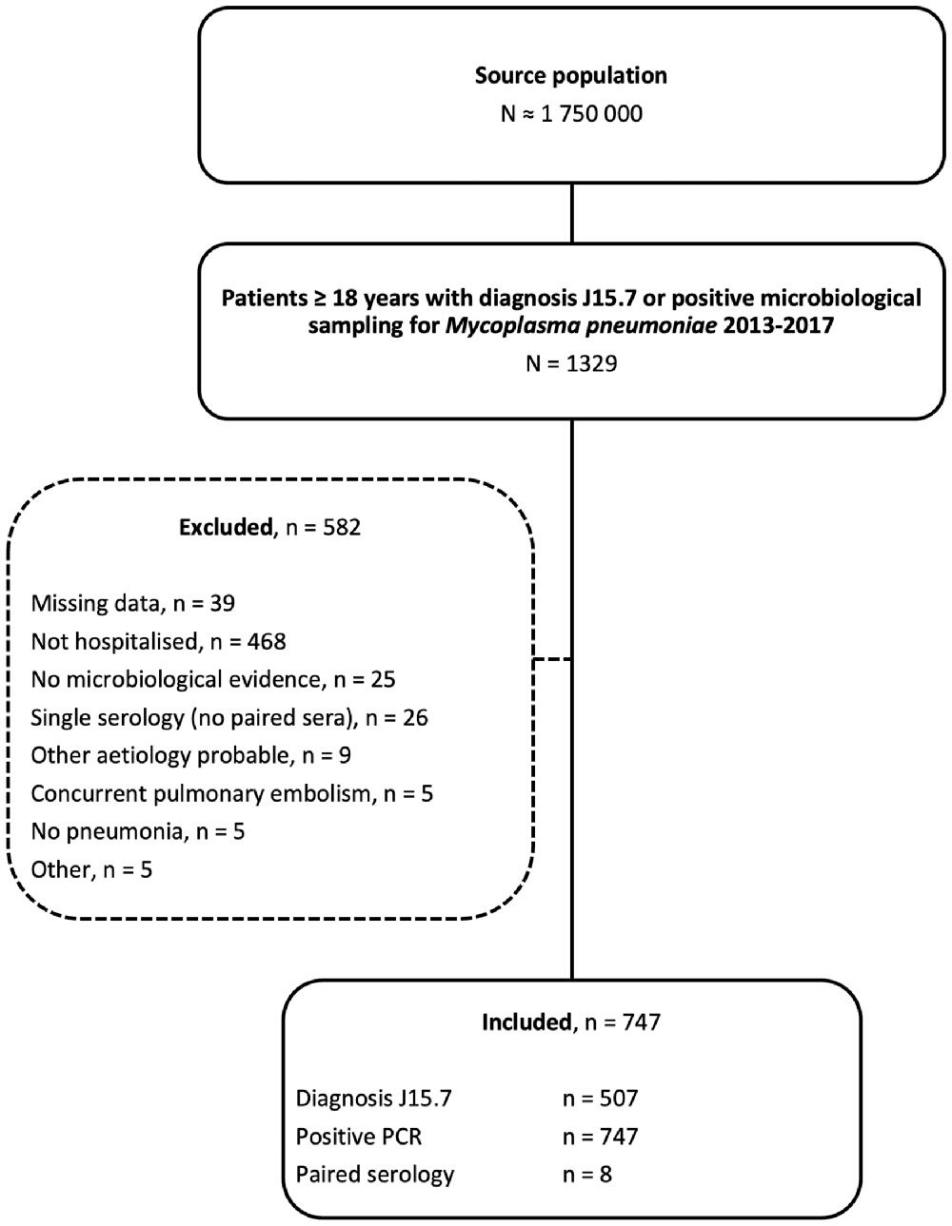
Flowchart of study population. Abbreviation: PCR, polymerase chain reaction.

A total of 747 patients were included in the cohort (Figure 1), with 62% (460/747) classified as having severe disease (estimated PaO_2_/FiO_2_ ≤300) at admission. Included patients had a positive *M pneumoniae* PCR from nasopharyngeal (46% [343/747]), oropharyngeal (31% [232/747]), sputum (21% [158/747]), and bronchoalveolar lavage sampling (1% [10/747]). Four patients (0.5%) had unknown localization of sampling. Serology was infrequently used and only 8 patients (1%) had a significant titer increase in paired sera, all of whom were also PCR positive. Baseline characteristics are summarized in Table 1 and Supplementary Table 1. The median age of the cohort was 42 years (interquartile range [IQR], 33–55 years), spanning from 18 to 90 years, and 52% (285/747) were male. A majority (56% [420/747]) of the patients had no comorbidities. Patients presenting with severe disease were significantly older (median age, 44 years [IQR, 35–57 years] vs 40 years [IQR, 29–51 years]; *P* < .001), more likely to be male (57% [261/460] vs 43% [124/287]; *P* < .001), and had a higher median body mass index (26 kg/m^2^ [IQR, 24–30  kg/m^2^] vs 25 kg/m^2^ [IQR, 22–28 kg/m^2^]; *P* < .001) compared to those with mild disease.

**Table 1.** Patient Characteristics

Characteristic	Full Cohort (n = 747)	Mild Disease^a^ (n = 287)	Severe Disease^a^ (n = 460)
Age, y, median (IQR)	42 (33–55)	40 (29–51)	44 (35–57)
Male sex	385/747 (52)	124/287 (43)	261/460 (57)
Smoking status			
Nonsmoker	379/524 (72)	135/177 (76)	244/347 (70)
Current smoker	54/524 (10)	16/177 (9)	38/347 (11)
Previous smoker	91/524 (17)	26/177 (15)	65/347 (19)
Comorbidities			
No comorbidity	420/747 (56)	179/287 (59)	250/460 (54)
COPD	13/747 (2)	3/287 (1)	10/460 (2)
Asthma	71/747 (10)	28/287 (10)	43/460 (9)
Other pulmonary disease	13/747 (2)	9/287 (3)	4/460 (1)
Cerebrovascular disease	16/747 (2)	7/287 (2)	9/460 (2)
Congestive heart failure	24/747 (3)	12/287 (4)	12/460 (3)
Liver disease	5/747 (1)	4/287 (1)	1/460 (0)
Chronic kidney disease	19/747 (3)	10/287 (3)	9/460 (2)
Neoplastic disease	31/747 (4)	12/287 (4)	19/460 (4)
Immunosuppression	40/747 (5)	17/287 (6)	23/460 (5)
BMI, kg/m^2^, median (IQR)	25.5 (23–29)	24.7 (22–27.7)	26.1 (23.6–29.7)
<18.5	12/547 (2)	7/217 (3)	5/330 (2)
18.5–24.9	227/547 (42)	108/217 (50)	119/330 (36)
25–30	195/547 (36)	67/217 (31)	128/330 (39)
>30	113/547 (21)	35/217 (16)	78/330 (24)

Data are presented as no./No. (%) unless otherwise indicated.

Abbreviations: BMI, body mass index; COPD, chronic obstructive pulmonary disease; IQR, interquartile range.

^a^Mild disease defined as estimated partial pressure of oxygen in arterial blood (PaO_2_)/fraction of inspired oxygen (FiO_2_) >300 and severe disease as estimated PaO_2_/FiO_2_ ≤300 at admission. Missing data are detailed in Supplementary Table 1.

Forty-three patients (6%) were found to have an alternative airway pathogen. The most common pathogens were *Haemophilus influenzae* (10 cases), *Streptococcus pneumoniae* (8 cases), and *Moraxella catarrhalis* (8 cases). Additionally, 11 patients had a viral coinfection including rhinovirus (5 cases), coronavirus (3 cases), and 1 patient each with respiratory syncytial virus, adenovirus, and influenza A virus.

### Incidence

**Figure 2.**
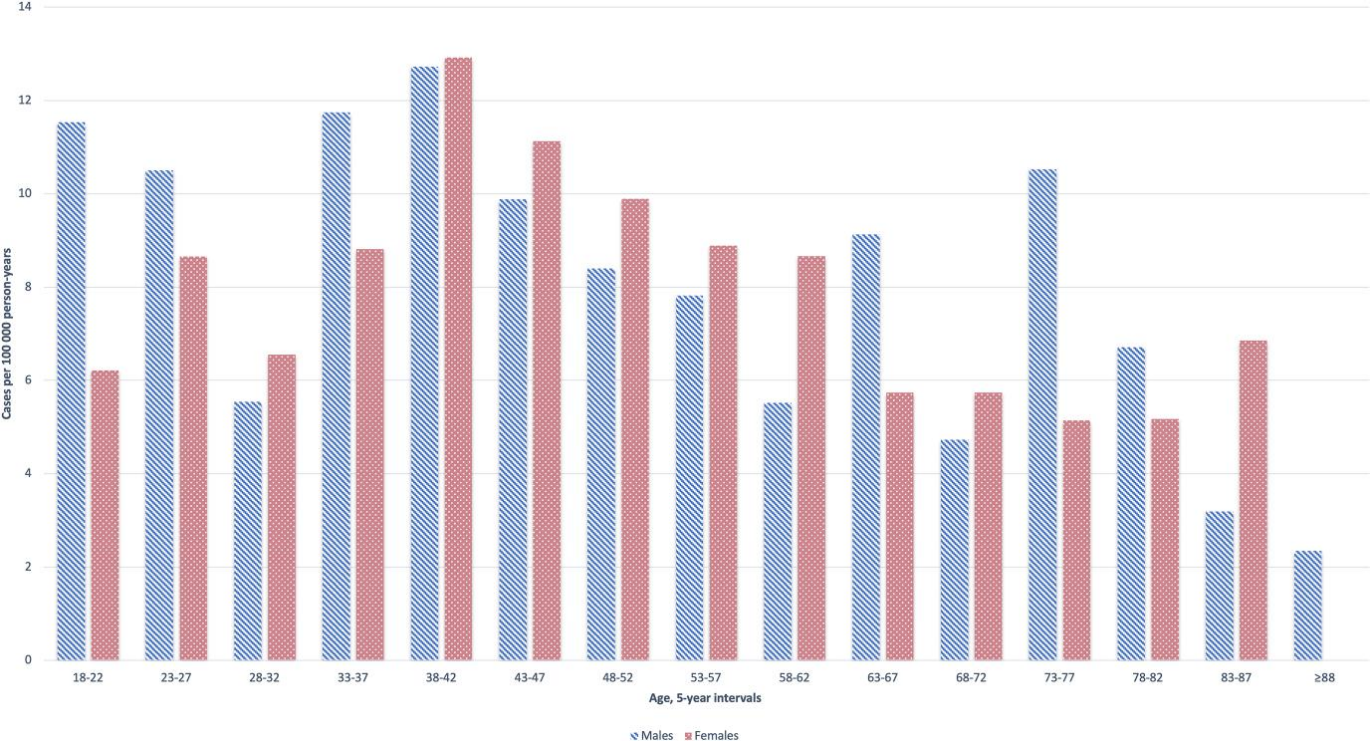
Incidence rate (cases per 100 000 person-years) of *Mycoplasma pneumoniae* pneumonia requiring hospitalization in adults by age and sex in Stockholm County, Sweden, 2013–2017.

**Figure 3.**
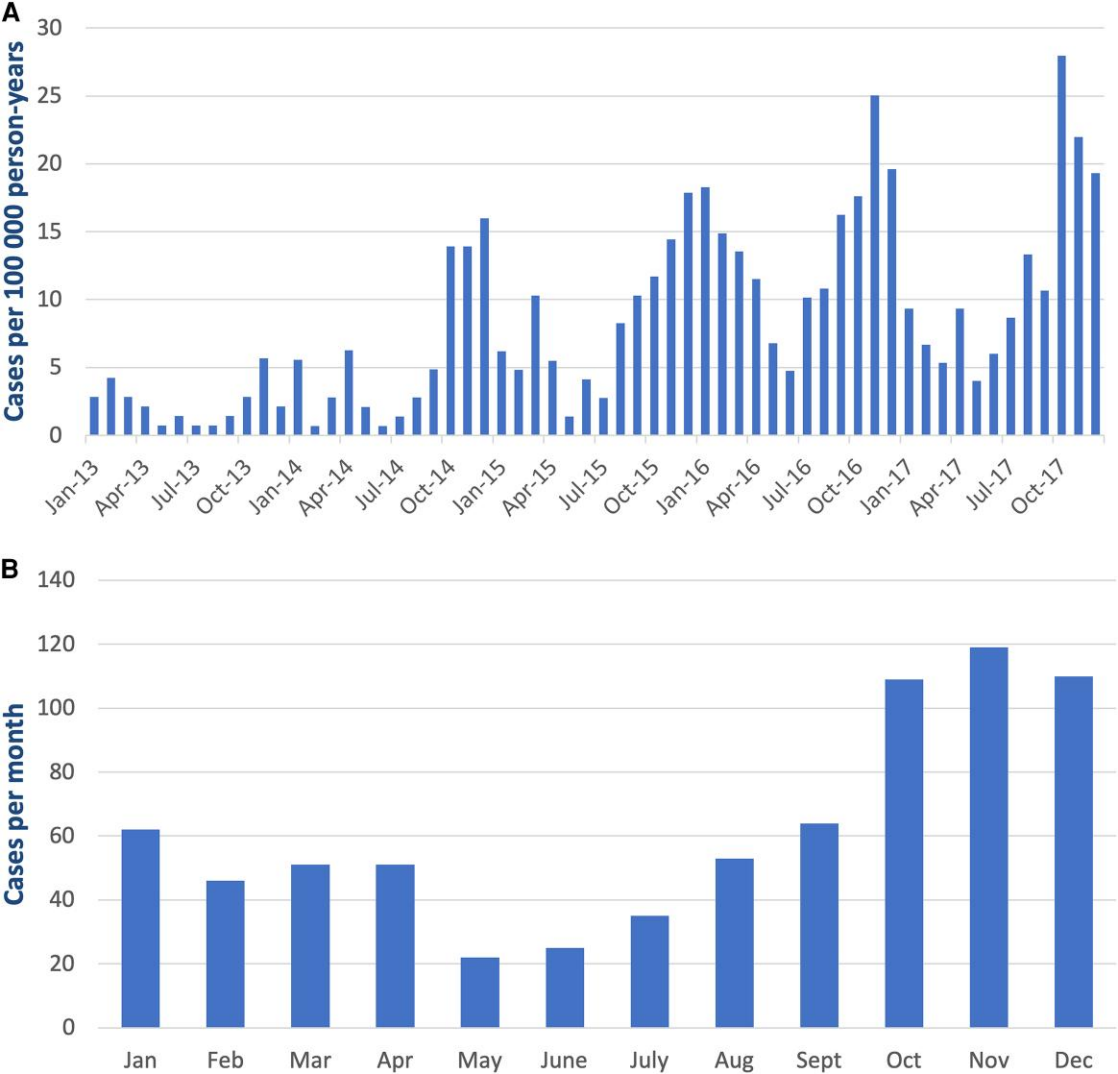
*A*, Incidence rate (cases per 100 000 person-years) of *Mycoplasma pneumoniae* pneumonia requiring hospitalization in adults per month in Stockholm County, Sweden, 2013–2017. *B*, Cumulative number of adults with *M pneumoniae* pneumonia needing hospitalization per calendar month in Stockholm County, Sweden, 2013–2017.

In Stockholm County, Sweden, the registered adult population increased from 1 697 119 inhabitants in 2013, to 1 802 149 inhabitants in 2017. During this period, the incidence rate of *M pneumoniae* pneumonia in adults needing hospitalization was 8.5 cases (95% CI, 7.9–9.2) per 100 000 PY. The incidence rate was 8.9 cases (95% CI, 8.1–9.9) per 100 000 PY for men and 8.2 cases (95% CI, 7.4–9.1) per 100 000 PY for women (*P* = .23). The incidence rates by sex and age are presented in Figure 2. The annual incidence rate varied (*P* < .001) from 2.3 cases (95% CI, 1.6–3.1) per 100 000 PY in 2013 to 14.1 cases (95% CI, 12.4–16.0) per 100 000 PY in 2016 (Figure 3*A*), with the highest cumulative monthly incidences occurring from October to December (Figure 3*B*).

When comparing cases of *M pneumoniae* pneumonia to the total number of patients hospitalized with a diagnosis of bacterial pneumonia in Stockholm County from 2013 to 2017, the proportion was 2.2% (747/33 908). This proportion ranged from 0.6% (39/6075) in 2013 to 3.4% (250/7322) in 2016.

### Clinical Presentation at Admission

Symptoms, clinical findings at examination, laboratory and radiological data, and risk scoring at admission are presented in Table 2 and Supplementary Table 2. The most commonly reported symptoms were cough (95%), fever (92%), and dyspnea (54%). Patients with severe disease had a significantly longer median symptom duration (8 days [IQR, 6–10 days]) compared to those with mild disease (6 days [IQR, 4–10 days]) (*P* < .001). In line with this, there was a significant but weak correlation (Spearman ρ = −0.23, *P* < .001) between estimated PaO_2_/FiO_2_ and symptom duration (Supplementary Figure 1), indicating that patients with longer symptom duration had lower estimated PaO_2_/FiO_2_ at admission. There was no significant difference in median estimated PaO_2_/FiO_2_ between patients admitted to different study hospitals.

**Table 2.** Clinical Presentation at Admission

Variable	Full Cohort (n = 747)	Mild Disease^a^ (n = 287)	Severe Disease^a^ (n = 460)
Patient history			
Symptom duration, d	7 (5–10)	6 (4–10)	8 (6–10)
Cough	708/747 (95)	265/287 (92)	443/460 (96)
Fever	215/233 (92)	69/74 (93)	146/159 (92)
Dyspnea	402/747 (54)	100/287 (35)	302/460 (66)
Fatigue	365/747 (49)	150/287 (52)	215/460 (47)
Productive cough	310/747 (42)	108/287 (38)	202/460 (44)
Headache	140/747 (19)	75/287 (26)	65/460 (14)
Gastrointestinal symptoms	46/247 (19)	24/82 (29)	22/165 (13)
Myalgia/arthralgia	126/747 (17)	53/287 (18)	73/460 (16)
Chest pain	86/747 (12)	34/287 (12)	52/460 (11)
Sore throat	63/747 (8)	26/287 (9)	37/460 (8)
Antibiotic treatment prior to admission	442/747 (59)	143/287 (50)	299/460 (65)
>1 antibiotic prior to admission	60/747 (8)	25/287 (9)	35/460 (8)
Effective antibiotic prior to admission	31/747 (4)	18/287 (6)	13/460 (3)
Findings at clinical examination			
Altered mental status (GCS score <15)	19/712 (3)	3/270 (1)	16/442 (4)
Body temperature, °C	38.7 (38.0–39.4)	38.7 (38.0–39.4)	38.6 (38.0–39.3)
Fever (≥38°C)	576/736 (78)	219/279 (78)	357/457 (78)
Heart rate, beats/min	104 (93–115)	102 (90–114)	106 (94–115)
Tachycardia (heart rate >100 beats/min)	466/732 (64)	156/276 (57)	310/456 (68)
Systolic BP, mm Hg	116 (106–127)	114 (105–123)	118 (107–129)
Diastolic BP, mm Hg	66 (60–73)	66 (60–72)	66 (60–74)
Hypotension (systolic BP <90 mm Hg)	16/735 (2)	8/278 (3)	8/457 (2)
Respiratory rate, breaths/min	24 (20–28)	21 (18–24)	26 (22–30)
Respiratory rate ≥30 breaths/min	164/729 (23)	25/277 (9)	139/452 (31)
Hypoxemia (SpO_2_ <93% or oxygen treatment)	522/740 (71)	62/280 (22)	460/460 (100)
Estimated PaO_2_/FiO_2_	286 (252–324)	343 (324–367)	262 (224–283)
Extrapulmonary manifestation	30/747 (4)	16/287 (6)	14/460 (3)
Laboratory and radiology investigatory results			
Lactate, mmol/L	1.1 (0.8–1.4)	1.1 (0.9–1.4)	1.1 (0.8–1.4)
Lactate ≥2 mmol/L	50/385 (13)	17/110 (15)	33/275 (12)
C-reactive protein, mg/L	170 (105–246)	147 (95–218)	190 (114–259)
Leukocyte count, ×10^9^/L	9.7 (7.6–12.3)	8.7 (6.7–10.9)	10.2 (8.2–13.2)
Platelet count, ×10^9^/L	279 (209–374)	243 (195–318)	298 (222–386)
Creatinine, µmol/L	72 (58–88)	71 (57–88)	72 (59–88)
Lactate dehydrogenase, µkat/L	4.0 (3.4–5.8)	3.5 (3.0–3.9)	4.8 (3.7–6.4)
Infiltrates on X-ray or CT scan	738/745 (99)	284/287 (99)	454/458 (99)
Bilateral pneumonia on X-ray or CT scan	362/744 (49)	92/286 (32)	270/458 (59)
Pleural effusion on X-ray or CT scan	153/744 (21)	57/286 (20)	96/458 (21)
Coinfection/colonization	45/747 (6)	17/287 (6)	28/460 (6)
*Mycoplasma pneumoniae* PCR Ct value	29.1 (22.6–34.5)	30.3 (23.4–35.0)	28.3 (22.4–33.9)
Risk scoring			
PSI points	53 (39–73)	43 (31–58)	61 (46–79)
PSI risk class I	149/747 (20)	112/287 (39)	37/460 (8)
PSI risk class II	391/747 (52)	132/287 (46)	259/460 (56)
PSI risk class III	114/747 (15)	25/287 (9)	89/460 (19)
PSI risk class IV	77/747 (10)	15/287 (5)	62/460 (13)
PSI risk class V	16/747 (2)	3/287 (1)	13/460 (3)

Data are presented as median (interquartile range) or no./No. (%).

Abbreviation: BP, blood pressure; Ct, cycle threshold; CT, computed tomography; FiO_2_, fraction of inspired oxygen; GCS, Glasgow Coma Scale; PaO_2_, partial pressure of oxygen in arterial blood; PCR, polymerase chain reaction; PSI, Pneumonia Severity Index; SpO_2_, peripheral oxygen saturation.

^a^Mild disease defined as estimated PaO_2_/FiO_2_ >300 and severe disease as estimated PaO_2_/FiO_2_ ≤300 at admission. Missing data are detailed in Supplementary Table 2.

Antibiotic treatments prior to admission were prescribed to 59% (442/747) of patients, and 8% (60/747) received multiple courses of antibiotics. Ineffective antibiotics were prescribed to 93% (411/442) of patients receiving treatment prior to admission, of which β-lactam antibiotics constituted 407 cases. A significantly higher proportion of patients with severe disease had received ineffective antibiotic treatment prior to admission (62% [286/460]) compared to those with mild disease (44% [125/287]) (*P* = .02). Median symptom duration was significantly longer (*P* < .001) in patients receiving antibiotics prior to admission (9 days [IQR, 7–11 days]) compared to those not receiving antibiotics prior to admission (5 days [IQR, 3–7 days]).

Extrapulmonary symptoms were reported in 30 patients (4%). Twelve patients (2%) had a skin rash, including 3 with erythema multiforme and 1 with vasculitis. Six patients (1%) had hemolytic anemia, and 5 (1%) had myocarditis/pericarditis. There was 1 patient each with iritis, conjunctivitis, Guillain–Barré syndrome, myocardial infarction, meningitis/myelitis, polyarthritis, and Stevens–Johnson syndrome. All patients with extrapulmonary infection received effective antibiotic treatment during hospitalization, and 11 of 30 (37%) received adjunctive corticosteroid treatment.

Hypoxemia (SpO_2_ <93% or receiving oxygen treatment) occurred in 71% (522/740) of patients at admission and the median respiratory rate was 24 (IQR, 20–28) breaths per minute. Bilateral infiltrates on X-ray or computed tomography scan of the thorax were reported in 49% (362/744) of patients, and 21% (153/744) had a pleural effusion. Signs of severe disease such as altered mental status (3% [19/712)], hypotension (2% [16/735]), or elevated lactate concentrations (13% [50/385]) were infrequent. In line with this, 12% (93/747) were classified as Pneumonia Severity Index (PSI) risk class IV–V.

### Treatments and Outcomes

Treatments and outcomes are presented in Table 3. Twenty-eight patients (4%) did not receive any effective antibiotics either prior to or during admission. Six of these 28 patients (21%) received corticosteroid treatment. Treatment with effective antibiotics was associated with significantly longer time to fever regression (*P* = .003) and length of stay (*P* < .001), compared to those not receiving effective antibiotic treatment. There was no significant difference in estimated PaO_2_/FiO_2_ between patients receiving and not receiving effective antibiotic treatment.

**Table 3.** Treatments and Outcomes

Variable	Full Cohort (n = 747)	Mild Disease^a^ (n = 287)	Severe Disease^a^ (n = 460)
Days from admission to effective antibiotics	0.7 (0–2.0)	0.8 (0–2.0)	0.7 (0–1.9)
Initial effective antibiotic	719/747 (96)	272/287 (95)	447/460 (97)
Tetracyclines	313/719 (44)	149/272 (55)	164/447 (37)
Macrolides	178/719 (25)	56/272 (21)	122/447 (27)
Fluoroquinolones	228/719 (32)	67/272 (25)	161/447 (36)
Duration of effective antibiotics, d	8 (7–10)	8 (7–10)	8 (7–10)
Corticosteroid treatment^b^	176/747 (24)	35/287 (12)	141/460 (31)
Corticosteroid dose, mg^c^	15 (9–20)	12 (6–19)	15 (9–20)
Duration of corticosteroid treatment, d	5 (3–6)	5 (2–5)	5 (3–7)
Duration of fever, d	2 (1–3)	1 (1–3)	2 (1–3)
ICU care	42/747 (6)	5/287 (2)	37/460 (8)
Duration of ICU care, d	2 (1–5)	1 (1–1)	3 (1–5)
In-hospital mortality	3/747 (0.4)	0/287 (0)	3/460 (1)
Length of stay, d	4 (2–6)	3 (2–5)	4 (3–7)

Data are presented as median (interquartile range) or no./No. (%).

Abbreviation: ICU, intensive care unit.

^a^Mild disease defined as estimated partial pressure of oxygen in arterial blood (PaO_2_)/fraction of inspired oxygen (FiO_2_) >300 and severe disease as estimated PaO_2_/FiO_2_ ≤300 at admission.

^b^Receipt of at least 1 oral or intravenous glucocorticoid dose.

^c^Median cumulative betamethasone equivalent dose.

Initial effective antibiotics during hospitalization were tetracyclines in 44% (313/719), macrolides in 25% (178/719), and fluoroquinolones in 32% (228/719) of patients. Patients with severe disease more often (*P* < .001) received treatment with macrolides and fluoroquinolones compared to those with mild disease. In line with this, median estimated PaO_2_/FiO_2_ was significantly lower in patients receiving fluoroquinolones (266 [IQR, 232–310]) as initial effective antibiotics compared to those receiving macrolides (280 [IQR, 243–310]) or tetracyclines (300 [IQR, 267–343]) (*P* < .001). Additionally, the choice of first-line effective treatment varied between the 7 hospitals in the region (Supplementary Table 3).

Doxycycline (313/313) was the only tetracycline used. Patients treated with macrolides received erythromycin (92% [164/178]) or azithromycin (8% [14/178]). Of those treated with fluoroquinolones, 55% (125/228) received levofloxacin, 45% (102/228) moxifloxacin, and 0.4% (1/228) ciprofloxacin.

Twenty-three percent (165/719) of patients receiving effective antibiotics switched to another antibiotic class during hospitalization. Changing to another antibiotic class was most common in patients receiving initial treatment with macrolides (39% [70/178]), followed by fluoroquinolones (31% [70/228]) and tetracyclines (8% [25/313]) (*P* < .001). Two-thirds (110/165) of these patients received a tetracycline as a secondary effective antibiotic. Tetracyclines were thus the most common antibiotics (57% [337/656]) for patients after discharge compared to macrolides (14% [92/656]) and fluoroquinolones (29% [187/656]).

**Figure 4.**
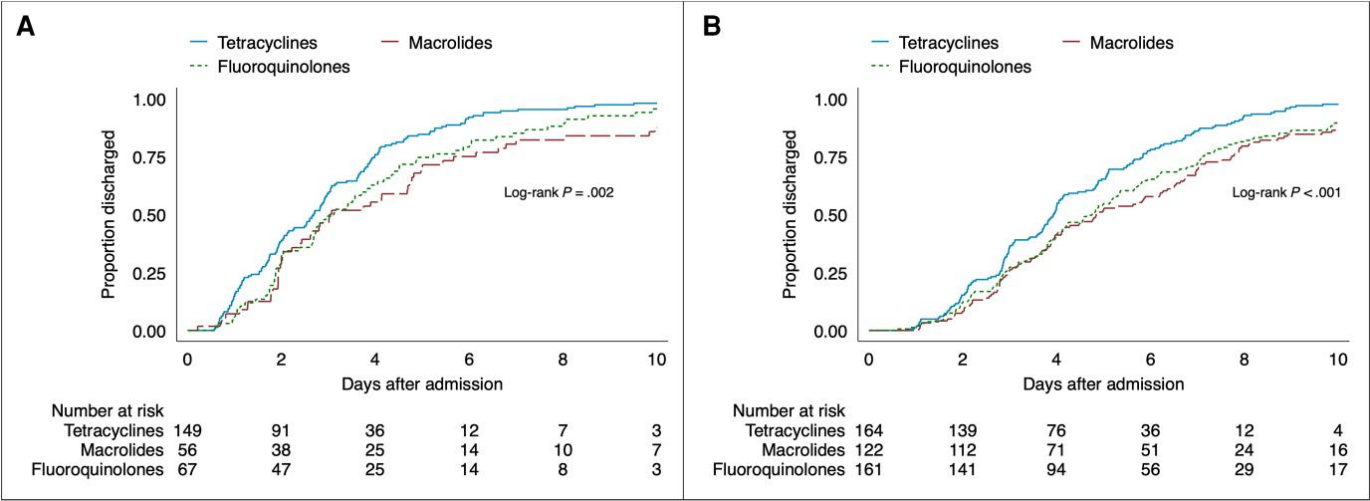
Kaplan-Meier curves of length of stay for patients with mild (estimated partial pressure of oxygen in arterial blood [PaO_2_]/fraction of inspired oxygen [FiO_2_] >300) (*A*) and severe (estimated PaO_2_/FiO_2_ ≤300) (*B*) disease at admission receiving different types of initial effective antibiotics during hospitalization. The log-rank test evaluates the equality of the survivor function across groups.

The median length of stay was 4 days (IQR, 2–6 days) in the full cohort. Length of stay was significantly longer in patients receiving treatment with macrolides (+1.0 days [IQR, 0.9–1.2 days]; *P* < .001) and fluoroquinolones (+0.8 days [IQR, 0.1–1.4 days]; *P* = .03) as initial effective antibiotics compared to those receiving tetracyclines, in a median regression adjusted for age, sex, estimated PaO_2_/FiO_2_, symptom duration, corticosteroid treatment, and time from admission to effective antibiotics (Supplementary Table 4). Kaplan-Meier curves of length of stay in patients receiving different types of antibiotics showed a higher discharge rate in patients receiving tetracyclines with similar results both in patients with mild disease and those with severe disease at admission (Figure 4). Moreover, patients with lower estimated PaO_2_/FiO_2_, higher age, longer time from admission to effective antibiotics, and corticosteroid treatment had significantly longer median length of stay in the multivariable regression (Supplementary Table 4). Results were consistent in a sensitivity analysis including only patients who did not switch to another antibiotic class during hospitalization (data not shown).

The median duration of fever was 2 days (IQR, 1–3 days) in patients with fever at admission (n = 567). Fever duration was significantly longer (+0.3 days [IQR, 0.1–0.6 days]; *P* = .02) in patients receiving fluoroquinolones compared to those receiving tetracyclines, in a median regression adjusted for age, sex, estimated PaO_2_/FiO_2_, symptom duration, corticosteroid treatment, and time from admission to effective antibiotics (Supplementary Table 5). Fever duration did not differ significantly between patients receiving macrolides and tetracyclines. Patients with higher age and longer time from admission to effective antibiotics had longer fever duration, and patients with longer symptom duration had shorter median fever duration in the multivariable regression (Supplementary Table 5). Results were mostly consistent in a sensitivity analysis including only patients who did not switch to another antibiotic class during hospitalization (data not shown).

In-hospital mortality was 0.4% (3/747), and 6% (42/747) of patients were admitted to an intensive care unit (ICU). The 3 patients who died during hospitalization had significant comorbidities, including neoplasia, Down syndrome, and prior cerebrovascular lesions. Two were elderly (>80 years old) and only 1 was admitted to an ICU. All were classified as having severe disease at admission. Among patients with mild disease at admission, 2% (5/287) deteriorated during hospitalization and required ICU care.

## DISCUSSION

This retrospective cohort study (N = 747) provides a comprehensive analysis of the incidence, patient characteristics, treatments, and outcomes of adults hospitalized with *M pneumoniae* pneumonia in a Swedish region over a 5-year period after the introduction of NAAT-based diagnostic testing. The findings highlight several aspects of *M pneumoniae* pneumonia, contributing to the broader understanding of its epidemiology and clinical management.

### Incidence

The incidence rate of *M pneumoniae* pneumonia in this study was 8.5 cases per 100 000 PY, representing 2.2% of all adult patients hospitalized with pneumonia. The incidence rate and prevalence are consistent with previous reported findings where *M pneumoniae* contributed to 2% (43/2272) of adults hospitalized with CAP, representing an incidence rate of 5 cases (95% CI, 4–7) per 100 000 PY [4]. The notable annual variation, with a peak incidence in 2016, is in line with the known cyclical epidemic pattern of *M pneumoniae* infections, which typically exhibit peaks every 4 years [2]. This pattern is also consistent with previous data indicating an epidemic in Europe during the winter seasons of 2014–2015 and 2015–2016 [8]. These results underscore the importance of heightened clinical and diagnostic vigilance during epidemic periods.

### Patient Characteristics and Clinical Presentation

The cohort predominantly comprised younger adults with a median age of 42 years, and a significant proportion had no comorbidities. This demographic profile is consistent with earlier studies indicating that *M pneumoniae* pneumonia often affects younger, healthier individuals compared to CAP of other etiologies [6, 16]. However, a relatively high proportion of older patients were included, with 14% (106/747) being 65 years or older. This age distribution is similar to previous Swedish data but differs from most other European countries, where *M pneumoniae* infections are infrequently diagnosed in older individuals [8]. This finding suggests that *M pneumoniae* infections occur in older patients and may be underdiagnosed.

A high proportion of patients had severe respiratory illness, but few had signs of systemic infection such as hypotension or elevated lactate concentrations. Patients with severe disease were older, had higher body mass index, and were more often male, suggesting that these factors may contribute to disease severity. Additionally, patients with severe disease more often received ineffective antibiotic treatment prior to admission and had longer symptom durations. This highlights the risk of delayed diagnosis and a need for improved diagnostic protocols to ensure timely and appropriate treatment.

### Treatments and Outcomes

Unexpectedly, patients receiving no effective treatment during hospitalization had better outcomes. This group likely represent patients where the microbiological results were received after discharge. However, follow-up data were not available from after discharge.

Hospital discharge occurred approximately 1 day faster in patients treated with tetracyclines compared to those treated with macrolides or fluoroquinolones even after adjusting for confounders such as disease severity. The choice of first-line antibiotic treatment varied due to local traditions rather than patient disease severity across different hospitals in the region. This strengthens the findings, although residual confounding such as disease severity may still be present. These results differ from previous retrospective studies that reported no significant differences in length of stay or fever duration between these antibiotic classes [11, 17]. The prevalence of macrolide-resistant *M pneumoniae* in Sweden 2016–2017 was reported to be 2% (1/61), and no clinical isolates with fluoroquinolone resistance have been identified, indicating that antibiotic resistance did not affect outcomes [9, 18]. These results suggest that doxycycline is an effective first-line treatment option for adults hospitalized with *M pneumoniae* pneumonia.

The low in-hospital mortality rate (0.4%) and ICU admission rate (6%) reflect the generally favorable outcomes for *M pneumoniae* pneumonia. However, the significant morbidity associated with severe disease underscores the need for early and effective treatment.

### Strengths and Limitations

The strengths of this study include the comprehensive inclusion of all diagnosed patients from a well-defined source population, detailed clinical data, and the use of reliable population denominator data from Swedish statistics databases. The retrospective design may introduce selection bias, and the reliance on medical records for data extraction could result in misclassification or incomplete data. Additionally, the study was conducted in a single geographic region, which may limit the generalizability of the findings to other settings. Despite using multivariable models to analyze the correlation of antibiotic class to outcomes, there remains a risk of residual confounding by indication, as patients treated with tetracyclines more often had mild disease. Moreover, there is a possibility of information bias in time to regression of fever as different hospitals may have had varying precision of temperature data. Future prospective studies are needed to validate these results and explore the long-term outcomes of *M pneumoniae* pneumonia.

## CONCLUSIONS

This study of 747 adults hospitalized with *M pneumoniae* pneumonia provides valuable insights into the epidemiology, clinical characteristics, and management of this disease. The findings highlight the importance of timely and accurate treatment and potential benefits of doxycycline as a first-line treatment. These insights can inform clinical practice and guide future research to improve the care of patients with *M pneumoniae* pneumonia.

## Supplementary Material

ofaf380_Supplementary_Data

## Supplementary Data

Supplementary materials are available at *Open Forum Infectious Diseases* online. Consisting of data provided by the authors to benefit the reader, the posted materials are not copyedited and are the sole responsibility of the authors, so questions or comments should be addressed to the corresponding author.
