# Supplementary material for: Epidemiology, Characteristics, and Treatment Outcomes of Mycoplasma pneumoniae Pneumonia in Hospitalized Adults: A 5-Year Retrospective Cohort Study
Source: Open Forum Infect Dis. 2025 Jun 26;12(7):ofaf380. doi: 10.1093/ofid/ofaf380 (PMC12308177; doi:10.1093/ofid/ofaf380)
Supplement: ofaf380_Supplementary_Data [file ofaf380_supplementary_data.docx]

# Supplementary Material

## Supplementary Table 1

| Supplementary Table 1. Missing Data for Table 1, Patient Characteristics | | | |
| --- | --- | --- | --- |
| *Demographics* | ***Full cohort***  ***(n = 747)*** | ***Mild disease***  ***(n =287)*** | ***Severe disease***  ***(n = 460)*** |
| Age | 0 (0) | 0 (0) | 0 (0) |
| Sex | 0 (0) | 0 (0) | 0 (0) |
| Smoking status | 223 (30) | 110 (38) | 113 (25) |
| Comorbidities | 0 (0) | 0 (0) | 0 (0) |
| Body Mass Index | 200 (27) | 70 (24) | 130 (28) |
| *Number of patients (%) with missing data for variables in Table 1.* | | | |

## Supplementary Table 2

| Supplementary Table 2. Missing Data for Table 2, Clinical Presentation at Admission | | | |
| --- | --- | --- | --- |
| *Variable* | ***Full cohort***  ***(n = 747)*** | ***Mild disease***  ***(n = 287)*** | ***Severe disease***  ***(n = 460)*** |
| Patient History |  |  |  |
| Symptom duration | 14 (2) | 8 (3) | 6 (1) |
| Cough | 79 (11) | 22 (8) | 17 (4) |
| Fever | 514 (69) | 213 (74) | 301 (65) |
| Dyspnoea | 0 (0) | 0 (0) | 0 (0) |
| Fatigue | 0 (0) | 0 (0) | 0 (0) |
| Productive cough | 0 (0) | 0 (0) | 0 (0) |
| Headache | 0 (0) | 0 (0) | 0 (0) |
| Gastrointestinal symptoms | 500 (67) | 205 (71) | 295 (64) |
| Myalgia/arthralgia | 0 (0) | 0 (0) | 0 (0) |
| Chest pain | 0 (0) | 0 (0) | 0 (0) |
| Sore throat | 0 (0) | 0 (0) | 0 (0) |
| Antibiotic treatment prior to admission | 0 (0) | 0 (0) | 0 (0) |
| Findings at Clinical Examination |  |  |  |
| Altered mental status | 35 (5) | 17 (6) | 18 (4) |
| Body temperature | 11 (1) | 8 (3) | 3 (1) |
| Heart rate | 15 (2) | 11 (4) | 4 (1) |
| Blood pressure | 12 (2) | 9 (3) | 3 (1) |
| Respiratory Rate | 18 (2) | 10 (3) | 8 (2) |
| Estimated PaO_2_/FiO_2_ | 7 (1) | 7 (2) | 0 (0) |
| Extrapulmonary manifestation | 0 (0) | 0 (0) | 0 (0) |
| Laboratory and Radiology Investigatory Results |  |  |  |
| Lactate | 362 (48) | 177 (62) | 185 (40) |
| C-reactive protein | 3 (0.4) | 1 (0.3) | 2 (0.4) |
| Leukocyte count | 25 (3) | 7 (2) | 18 (4) |
| Platelet count | 116 (16) | 56 (20) | 60 (13) |
| Creatinine | 54 (7) | 19 (7) | 35 (8) |
| Lactate dehydrogenase | 676 (90) | 261 (91) | 415 (90) |
| X-ray or computed tomography scan | 3 (0.4) | 1 (0.3) | 2 (0.4) |
| Co-infection/Colonisation | 0 (0) | 0 (0) | 0 (0) |
| *M. pneumoniae* PCR Cycle Threshold-value | 377 (50) | 149 (52) | 228 (50) |
| Risk Scoring |  |  |  |
| Pneumonia Severity Index | 0 (0) | 0 (0) | 0 (0) |
| *Number of patients (%) with missing data for variables in Table 1.* | | | |

## Supplementary Table 3

| Supplementary Table 3. Number of Patients (%) Treated with Different Antibiotics at Different Hospitals | | | | | | | |
| --- | --- | --- | --- | --- | --- | --- | --- |
| Antibiotic treatment | **Hospital 1** | **Hospital 2** | **Hospital 3** | **Hospital 4** | **Hospital 5** | **Hospital 6** | **Hospital 7** |
| Tetracyclines | 44 (33) | 8 (31) | 5 (36) | 33 (36) | 45 (32) | 87 (59) | 91 (56) |
| Macrolides | 78 (58) | 5 (19) | 5 (36) | 29 (32) | 26 (18) | 26 (18) | 9 (5) |
| Fluoroquinolones | 12 (9) | 13 (50) | 4 (29) | 30 (33) | 70 (50) | 35 (24) | 64 (39) |
| Total | 134 (100) | 26 (100) | 14 (100) | 92 (100) | 141 (100) | 148 (100) | 164 (100) |

## Supplementary Table 4

| Supplementary Table 4. Median Length of Stay | | | | | | | | |
| --- | --- | --- | --- | --- | --- | --- | --- | --- |
|  | **Univariate** | | | | **Multivariate** | | | |
| Variable | | **β** | **95% CI** | ***P* value** | | **β** | **95% CI** | ***P* value** |
| Constant | |  |  |  | | 3.45 | 2.02-4.89 | < 0.001 |
| Estimated PaO_2_/FiO_2_ | | -0.01 | -0.01--0.01 | < 0.001 | | -0.01 | -0.01--0.01 | < 0.001 |
| Age (years) | | 0.06 | 0.04-0.07 | < 0.001 | | 0.02 | 0.01-0.04 | < 0.001 |
| Male sex | | 0.17 | -0.49-0.84 | 0.61 | | -0.21 | -0.45-0.04 | 0.10 |
| Symptom duration (days) | | -0.02 | -0.07-0.04 | 0.54 | | -0.02 | -0.04-0.01 | 0.08 |
| Time from admission to effective antibiotics (days) | | 0.86 | 0.67-1.05 | < 0.001 | | 0.84 | 0.70-0.97 | < 0.001 |
| Corticosteroid treatment | | 2.91 | 2.23-3.59 | < 0.001 | | 1.05 | 0.33-1.77 | 0.004 |
| Initial effective antibiotic class | |  |  |  | |  |  |  |
| Tetracyclines | | Ref. | Ref. | Ref. | | Ref. | Ref. | Ref. |
| Macrolides | | 1.37 | 1.01-1.72 | < 0.001 | | 1.04 | 0.92-1.17 | < 0.001 |
| Fluoroquinolones | | 1.07 | -0.25-2.39 | 0.11 | | 0.75 | 0.06-1.44 | 0.03 |
| Median regression model of length of stay with 95 % Confidence intervals (95% CI). Severe disease defined as estimated PaO_2_/FiO_2_ ≤ 300 at admission. | | | | | | | | |

## Supplementary Table 5

| Supplementary Table 5. Median Duration of Fever | | | | | | | | |
| --- | --- | --- | --- | --- | --- | --- | --- | --- |
|  | **Univariate** | | | | **Multivariate** | | | |
| Variable | | **β** | **95% CI** | ***P* value** | | **β** | **95% CI** | ***P* value** |
| Constant | |  |  |  | | 1.14 | -0.12-2.40 | 0.08 |
| Estimated PaO_2_/FiO_2_ | | 0.00 | -0.00-0.00 | 0.77 | | -0.00 | -0.00-0.00 | 0.78 |
| Age (years) | | 0.02 | 0.01-0.02 | < 0.001 | | 0.01 | 0.00-0.01 | 0.04 |
| Male sex | | -0.02 | -0.26-0.22 | 0.85 | | -0.03 | -0.41-0.34 | 0.87 |
| Symptom duration (days) | | -0.09 | -0.12- -0.06 | < 0.001 | | -0.04 | -0.05--0.02 | < 0.001 |
| Time from admission to effective antibiotics (days) | | 0.67 | 0.56.0.78 | < 0.001 | | 0.64 | 0.51-0.76 | < 0.001 |
| Corticosteroid treatment | | -0.02 | -0.48-0.44 | 0.92 | | -0.09 | -0.48-0.31 | 0.67 |
| Initial effective antibiotic class | |  |  |  | |  |  |  |
| Tetracyclines | | Ref. | Ref. | Ref. | | Ref. | Ref. | Ref. |
| Macrolides | | 0.09 | -0.34-0.53 | 0.67 | | -0.09 | -0.39-0.20 | 0.53 |
| Fluoroquinolones | | 0.40 | -0.04-0.83 | 0.07 | | 0.34 | 0.05-0.62 | 0.02 |
| Median regression model of duration of fever with 95 % Confidence intervals (95% CI). Severe disease defined as estimated PaO_2_/FiO_2_ ≤ 300 at admission. | | | | | | | | |

Supplementary Figures





**Supplementary Figure 1**. Scatterplot of estimated PaO_2_/FiO_2_ and symptom duration at admission.

**Alt text supp 1:** Scatterplot of estimated PaO_2_/FiO_2_ and symptom duration at admission.
